# The G protein-coupled receptor subset of the dog genome is more similar to that in humans than rodents

**DOI:** 10.1186/1471-2164-10-24

**Published:** 2009-01-15

**Authors:** Tatjana Haitina, Robert Fredriksson, Steven M Foord, Helgi B Schiöth, David E Gloriam

**Affiliations:** 1 Department of Neuroscience, Functional Pharmacology, Uppsala University, BMC, Box 593, 751 24, Uppsala, Sweden; 2 GlaxoSmithKline Pharmaceuticals, New Frontiers Science Park, 3rd Avenue, Harlow CM19 5AW, UK

## Abstract

**Background:**

The dog is an important model organism and it is considered to be closer to humans than rodents regarding metabolism and responses to drugs. The close relationship between humans and dogs over many centuries has lead to the diversity of the canine species, important genetic discoveries and an appreciation of the effects of old age in another species. The superfamily of G protein-coupled receptors (GPCRs) is one of the largest gene families in most mammals and the most exploited in terms of drug discovery. An accurate comparison of the GPCR repertoires in dog and human is valuable for the prediction of functional similarities and differences between the species.

**Results:**

We searched the dog genome for non-olfactory GPCRs and obtained 353 full-length GPCR gene sequences, 18 incomplete sequences and 13 pseudogenes. We established relationships between human, dog, rat and mouse GPCRs resolving orthologous pairs and species-specific duplicates. We found that 12 dog GPCR genes are missing in humans while 24 human GPCR genes are not part of the dog GPCR repertoire. There is a higher number of orthologous pairs between dog and human that are conserved as compared with either mouse or rat. In almost all cases the differences observed between the dog and human genomes coincide with other variations in the rodent species. Several GPCR gene expansions characteristic for rodents are not found in dog.

**Conclusion:**

The repertoire of dog non-olfactory GPCRs is more similar to the repertoire in humans as compared with the one in rodents. The comparison of the dog, human and rodent repertoires revealed several examples of species-specific gene duplications and deletions. This information is useful in the selection of model organisms for pharmacological experiments.

## Background

The dog is an important model in biomedical research for several reasons. Dogs have unique evolutionary history. Since their domestication from the grey wolf in East Asia about 100 000 years ago dogs have shared living space and food sources with humans and have been selectively inbred with periodic population bottlenecks [1,2]. The American Kennel Club (AKC) and similar organizations worldwide have provided easily accessible and extensive genealogies which provide unique opportunities for genetic analyses. Dogs show a high prevalence of specific diseases; such as blindness, heart disease, cataracts, epilepsy and deafness; that are relevant for human biology [3,4]. Dogs are susceptible to a wide variety of genetic diseases. For example, dogs have cancers that seem to affect just one breed or a few closely related breeds. The dog also has more similarities in general physiology, anatomy, disease susceptibility, morphological variation and behavioural traits to humans than the most frequently used experimental animals, mouse and rat. Regulatory authorities mandate the use of non-rodent species in safety assessments for new medicines and dogs are the most frequent choice. Furthermore, the dog is also an important model in evolutionary analysis in which its relative divergence in relation to other mammalian lineages allows for valuable comparisons. The sequence of the dog (*Canis familiaris*) genome has had contributions from two breeds, the boxer [5] and the poodle [6]. Analysis have revealed long-range haplotypes across the entire genomes, crucial for defining the nature of genetic diversity within and across breeds [5]. These maps provide good opportunities for genome-wide association studies to identify genes responsible for diseases and traits.

Automated gene predictions offer fast annotation of genomes but they are error-prone and need to be followed up by careful manual curation of the coding sequences. For instance the Genscan gene prediction program has a sensitivity and specificity of about 90% for detecting exons, leading to frequent errors in multi-exon genes [7]. Our recent annotation of the G protein-coupled receptors (GPCRs) within the chicken genome showed that over 60% of the Genscan gene predictions with a human ortholog needed curation. Curation markedly increased the quality of the dataset, raising the average percentage identity between the human-chicken one-to-one orthologous pairs from 56% to 73% [8]. The quality of protein sequences has a significant impact on phylogenetic analyses and calculations of evolutionary distances. Accurate comparisons of the dog and human proteins, such as correct assignment of orthologous pairs, are crucial for the design and interpretation of physiological and pharmacological studies in which results are inferred between the species.

The superfamily of GPCRs is one of the largest groups of proteins within most mammals. GPCRs are signal mediators that have a prominent role in most major physiological processes at both the central and peripheral level [9]. It has been estimated that about 80% of all known hormones and neurotransmitters activate cellular signal transduction mechanisms via GPCRs [10]. Many of GPCRs are able to form and function as heterodimers of two GPCR monomers (for example, GABA_B_R1-GABA_B_R2, TAS1R3-TAS1R1 and TAS1R3-TAS1R2) or even as heterodimers of a GPCR monomer and a receptor activity-modifying protein (RAMP) (for example, PTHR1-RAMP2) [11]. The key common structural components of the GPCRs are the seven transmembrane α-helices that span the cell membrane. GPCRs represent between 30-45% of the current drug targets [12,13] and many pharmaceutical companies devote up to 30% of their drug discovery efforts toward them [14]. Even so, they have an enormous unexploited therapeutic potential as drugs in the clinic target only 30 of the approximately 400 non-olfactory GPCRs [15].

The human GPCR repertoire has previously been divided into five main families (*GRAFS*); *Glutamate *(clan C), *Rhodopsin *(clan A, includes the olfactory receptors), *Adhesion *(clan B2), *Frizzled*/*Taste2 *and *Secretin *(clan B) [16]. The *GRAFS *families are found in all bilateral species [17]. The *Rhodopsin *family is the largest and includes hundreds of olfactory receptors (ORs). The *Rhodopsin *family also contains most of the GPCR drug targets, mainly amine and peptide receptors [18]. In humans, the second largest family is the *Adhesion *family. *Adhesion *GPCRs are characterized by long extracellular N-termini. Most of the receptors in this family are still orphans (i.e their endogenous ligands are unknown) [19,20] The *Glutamate *family includes receptors that are activated by glutamate, GABA and calcium as well as the two groups of sweet and umami taste receptors (TAS1Rs) and vomeronasal receptors type 2 (V2Rs) that recognize pheromones. The *Secretin *family has ligands that are large peptides such as secretin, parathyroid hormone, glucagon, glucagon-like peptide, calcitonin, vasoactive intestinal peptide, growth hormone releasing hormone and pituitary adenylyl cyclase activating protein. The *Frizzled *receptors bind, among others, the Wnt ligands and play an important role in embryonic development. The *Taste2 *or bitter taste receptor family was originally assigned together with Frizzled family, but they form two very distinct clusters [16]. It is not clear if *Frizzled *and *Taste2 *groups have a common evolutionary origin and in this study we describe them as two different families. Another family is the vomeronasal 1 receptors, abbreviated V1R, which does not display similarity to any of the GRAFS groups. V1R family has many members in rodents [21], but very few in humans and was therefore not included into the original GRAFS classification. A consensus list of all human, mouse and rat 'non sensory' GPCRs is maintained by IUPHAR [22]. The GPCR repertoire has also been studied in detail in non-mammalian vertebrates such as the teleost pufferfish [23] and in invertebrates such as the lancelet [24] and the mosquito [25].

The sense of smell, or odorant detection, is strongly evolved in dogs for which 876 genes have been predicted to encode olfactory receptors, a figure almost double the human repertoire and comparable to that of rodents [26]. The vomeronasal *V2R *receptors are also thought to serve an olfactory function [21], but surprisingly no such functional genes were found in dog, only pseudogenes. Rodents have a largely expanded *V1R *repertoire with over 100 genes, whereas dogs have 8 and humans have no *V1Rs *[21]. The dog also has 14 *Taste2 *receptors for bitter taste [27]. The non-sensory GPCRs have not previously been studied in dog.

In this study we provide the subset of the non-olfactory GPCRs in the dog genome. We have made comprehensive searches for dog GPCR genes, put extensive efforts in manually correcting coding sequences and performed detailed phylogenetic analyses. Furthermore, we provide a comparison between the GPCR repertoires in human, dog, mouse and rat.

## Results

We performed a comprehensive search for non-olfactory GPCR genes in the dog genome. A start dataset was produced from BLASTN searches in the Genbank non-redundant database. This contained 325 full-length GPCRs and 5 pseudogenes. Around 13% of these needed manual curation because they had an incorrect composition of exons. TBLASTN and BLAT searches in the dog genome assembly completed the analysis. A total number of 353 full-length sequences, 18 incomplete sequences and 13 pseudogenes were retrieved. A full-length dog GPCR gene has been defined as one that contains an intact transmembrane domain. The incomplete GPCR gene sequences are missing exons or parts thereof because they reside in genomic regions that have not been sequenced. It is also possible that whole GPCR genes are missing in the dog genome assembly and these can be very difficult to distinguish from those that do not exist in this species unless the specific genomic region is carefully analysed. The gene sequences of MAS1, NPY2R, GPR52 and GPR37L1 were found to include frameshifts and/or stop codons in the Broad Institute genome assembly (from the boxer). However, in a second BLAST search of these sequences in the TIGR poodle assembly [28] these 4 genes were found to be intact/full-length. This may either reflect sequencing issues or indicate real differences between breeds.

The dog GPCR gene sequences were divided into families in line with the *GRAFS *classification: *Glutamate*, *Rhodopsin, Adhesion*, *Frizzled, Secretin, Taste2 *and *V1R *families [29]. 18 genes do not have sequence similarity to any GPCR family and these were treated as a separate group called *Other GPCRs *according to our previous classification of the rat GPCRs [30], the only difference being that GPR149 was here moved to the *Rhodopsin *family. The numbers of genes in each GPCR family; including previously published sensory GPCRs for human, dog, mouse and rat; are presented in Table 1. A complete table of all *GRAFS *GPCR genes in dog, human, rat and mouse is presented in Additional file 1. The amino acid sequences of all dog GPCRs obtained in this study are included in Additional file 2.

**Table 1:** The number of GPCR genes in human, dog, mouse and rat.

**GPCR Family**	**Total Number of GPCRs**				
	Dog	Dog Pseudogenes	Human	Mouse	Rat

*Adhesion*	37	1	33	30	30
*Frizzled*	11	0	11	11	10
*Glutamate*	22	0	22	22	22
*Rhodopsin non-olfactory*	267	12	284	320	297
*Secretin*	15	0	15	15	15
*Taste 2*	14^#^	5^#^	25^§^	35^§^	35^§^
*V1R*	8*	33*	5*	187*	106*
*V2R*	0*	9*	0*	121*	79*
*Other GPCRs*	18	0	18	19	19

*[21]; # [27]; §[30]

**Figure 1:**
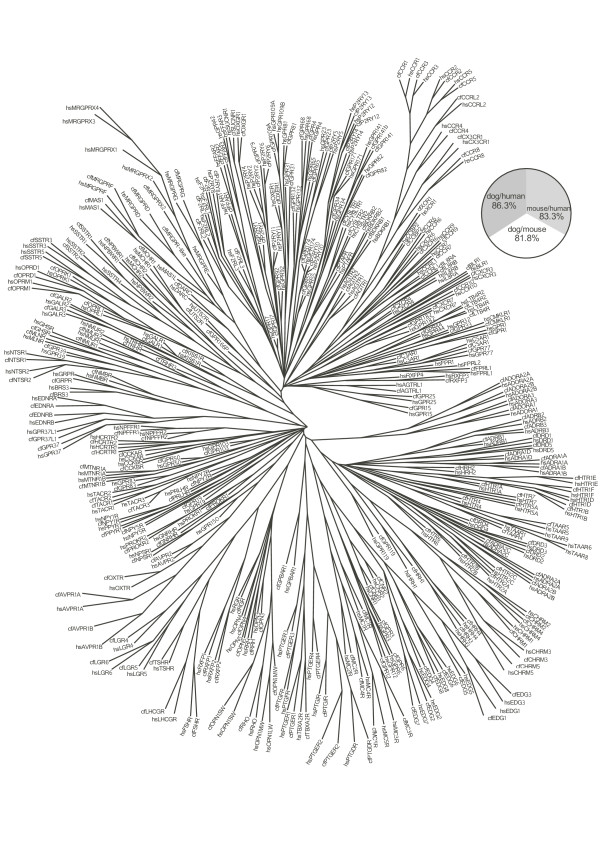
**Consensus tree of the human (hs) and dog (cf) *Rhodopsin *family based on 100 Maximum Parsimony phylogenetic trees**. The sequence alignment used for the phylogenetic calculation was based on the transmembrane segments. A pie-chart displays the average pairwise percentages of protein sequence identity between human, mouse and dog one-to-one orthologs.

**Figure 2:**
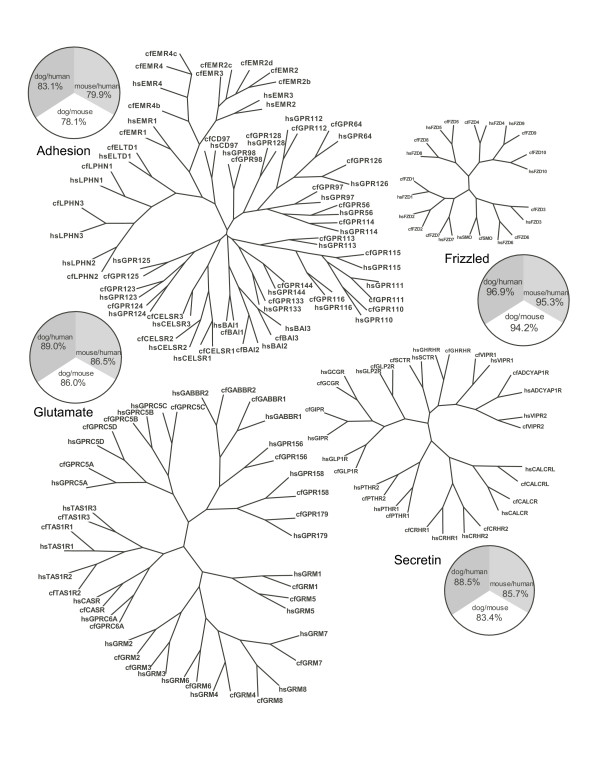
**Consensus trees of the human (hs) and dog (cf) *Adhesion*, *Frizzled*, *Glutamate *and *Secretin *GPCR families**. Each tree is based on 100 Maximum Parsimony trees. The sequence alignments used for phylogenetic calculations were based on the transmembrane segments. For each GPCR family a pie-chart displays the average pairwise percentages of protein sequence identity between human, mouse and dog one-to-one orthologs.

We performed phylogenetic analyses of all dog and human *GRAFS *GPCR protein sequences and identified orthologs and species-specific genes. The latter represent paralogous genes that have arisen or been lost specifically in either human or dog or the lineages leading to them. Consensus trees of 100 Maximum Parsimony phylogenetic trees and the average amino acid sequences identities of receptor orthologs are presented in Figure 1: *Rhodopsin *family and Figure 2:*Glutamate*, *Adhesion*, *Frizzled *and *Secretin *families. Dog genes missing in human are listed in Table 2, whereas human GPCR genes not found in the dog and/or rodent genomes are listed in Table 3.

**Table 2:** Table showing the dog GPCR genes that are missing or pseudogenes in human.

**Family**	**GPCR**	**Human**	**Dog**	**Rat**	**Mouse**
*Adhesion*	EMR2b	missing	present	missing	missing
*Adhesion*	EMR2c	missing	present	missing	missing
*Adhesion*	EMR2d	missing	present	missing	missing
*Adhesion*	EMR4b	missing	present	missing	missing
*Adhesion*	EMR4c	missing	present	missing	missing
*Rhodopsin*	GPR166P	pseudogene	present	present	pseudogene
*Rhodopsin*	GPR33	pseudogene	present	present	present
*Rhodopsin*	GPR79	pseudogene	present	present	present
*Rhodopsin*	TAAR4	pseudogene	present	present	present
*Rhodopsin*	GPR141b	missing	present	missing	missing
*Rhodopsin*	MRGPR-like1	missing	present	missing	missing
*Rhodopsin*	TRHR3	missing	present	missing	missing

**Table 3:** Human GPCR genes that are missing (not found in genome assemblies) or are pseudogenes in dog and/or rodents.

**Family**	**GPCR**	**Human**	**Dog**	**Rat**	**Mouse**
*Adhesion*	EMR2	present	present	missing	missing
*Adhesion*	EMR3	present	present	missing	missing
*Adhesion*	GPR144	present	present	pseudogene	pseudogene
*Rhodopsin*	AGTR1	present	present	missing	missing
*Rhodopsin*	CCR1	present	present	missing	missing
*Rhodopsin*	FPR1	present	missing	present	present
*Rhodopsin*	FPRL2	present	missing	missing	missing
*Rhodopsin*	GPR109B	present	missing	missing	missing
*Rhodopsin*	GPR135	present	missing	present	present
*Rhodopsin*	GPR148	present	missing	missing	missing
*Rhodopsin*	GPR150	present	missing	present	present
*Rhodopsin*	GPR32	present	missing	pseudogene	pseudogene
*Rhodopsin*	GPR42	present	missing	missing	missing
*Rhodopsin*	GPR75	present	missing	present	present
*Rhodopsin*	GPR78	present	pseudogene	missing	missing
*Rhodopsin*	HTR1E	present	present	missing	missing
*Rhodopsin*	MAS1L	present	missing	missing	missing
*Rhodopsin*	MCHR2	present	present	missing	missing
*Rhodopsin*	MLNR	present	present	missing	pseudogene
*Rhodopsin*	MRGPRE	present	missing	present	present
*Rhodopsin*	MRGPRX1	present	missing	missing	missing
*Rhodopsin*	MRGPRX2	present	present	missing	missing
*Rhodopsin*	MRGPRX3	present	missing	missing	missing
*Rhodopsin*	MRGPRX4	present	missing	missing	missing
*Rhodopsin*	NPBWR2	present	missing	pseudogene	pseudogene
*Rhodopsin*	OPN1LW	present	missing	missing	missing
*Rhodopsin*	OXER1	present	present	missing	missing
*Rhodopsin*	P2RY11	present	present	missing	missing
*Rhodopsin*	P2RY4	present	pseudogene	present	present
*Rhodopsin*	P2RY8	present	present	missing	missing
*Rhodopsin*	RXFP4	present	pseudogene	pseudogene	present
*Rhodopsin*	SSTR4	present	missing	present	present
*Rhodopsin*	TAAR1	present	pseudogene	present	present
*Rhodopsin*	TAAR6	present	missing	present	present
*Rhodopsin*	TAAR8	present	missing	missing	missing
*Rhodopsin*	TAAR9	present	missing	present	present

We identified 267 *Rhodopsin *GPCR genes in dog and this can be compared with the corresponding number in human that is 284 (Table 1 and Additional File 1). The average protein sequence identity is 86% between dog and human one-to-one orthologs and this is higher than is observed for each of these two species to the mouse orthologs. For ease of discussion we present the *Rhodopsin *family of GPCRs according to their broad phylogenetic grouping [16] (see Additional file 1).*Rhodopsin *α subfamily in dog is missing the receptors GPR148, Red opsin (OPN1LW), TAAR6, TAAR8 and TAAR9 (Table 3). In the rodent genomes, three of these receptors; GPR148, OPN1LW and TAAR8; are absent, whereas two; TAAR6 and TAAR9; are present. In dog GPR78 and TAAR1 are pseudogenes while TAAR4 is a full-length/intact gene in contrast to its human ortholog, which is a pseudogene. The gene sequences of dog ADRA1B, ADRA1D, ADRA2A, DRD4 and MTNR1B are incomplete.

In the *Rhodopsin *β subfamily one new dog gene, TRHR3, was identified. TRHR3 is not present in humans or rodents and the receptor with the highest amino acid identity, 59%, is the *Xenopus laevis *thyrotropin-releasing hormone receptor 3 (TRHR3, GenBank accession: CAD12656). Two *Rhodopsin *β subfamily receptors, GPR75 and GPR150, are missing in dog, but present in human and rodents. The dog NPFFR1 and NPFFR2 gene sequences are incomplete.

In the *Rhodopsin *γ subfamily the dog lacks the genes for FPR1, FPRL2, GPR32, NPBWR2 and SSTR4, which are all present in human (Table 3). GPR33, which is a pseudogene in human, is a full-length gene in both dog and rodents. In contrast, another gene, RXFP4, is a pseudogene in dog, but full-length in both human and rodents. The dog KISSR1 was found to have an incomplete sequence in the genome assembly.

In the *Rhodopsin *δ subfamily we identified one new dog member of the Mas-Related GPCR (MRG) cluster, MRGPR-like1. The dog assembly is missing, GPR109B, GPR42 (FFAR1L), MAS1L, MRGPRE, MRGPRX1, MRGPRX3 and MRGPRX4, which are all present in the human genome. GPR79 is a full-length gene in both dog and rodents, but is a pseudogene in human. In contrast, P2RY4 is a full-length gene in human and rodents, but not in dog in which it is a pseudogene. The dog MRGPRX2 gene sequence is incomplete.

One additional new dog *Rhodopsin *GPCR was identified, GPR141b. The most similar receptor, human GPR141, is an orphan GPCR. GPR135, which is also an orphan *Rhodopsin *GPCR, was not found in dog. GPR166P, which is a pseudogene in human, was found to be a full-length gene in dog appearing to be functional. Two additional dog *Rhodopsin *GPCRs, DARC and GPR88, have only incomplete gene sequences.

Figure 2 displays consensus trees of 100 maximum parsimony phylogenetic trees of the *Adhesion*, *Frizzled*, *Glutamate *and *Secretin *families of GPCRs. All families are relatively well conserved in terms of sequence identity (in the order *Frizzled *> *Glutamate *> *Secretin *> *Adhesion*).

The results show that the *Glutamate *family is well conserved having 22 orthologous receptor pairs and no species-specific genes in dog and human. (Figure 2 and Additional file 1). The average protein sequence identity is 89% between dog and human orthologs and lower for each of these two species to the mouse orthologs. The sequence of dog GRM3 is incomplete.

**Figure 3:**
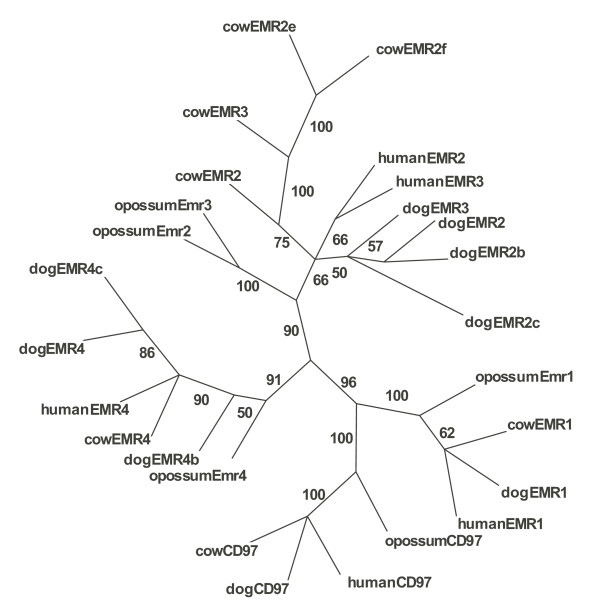
**Consensus tree of the EGF-TM7 *Adhesion *family GPCRs derived from 100 Maximum Parsimony phylogenetic trees**. The sequence alignment used for the phylogenetic calculation was based on the transmembrane segments.

The *Adhesion *family displays have unconventional orthology relationships between dog and human. All 33 human *Adhesion *GPCRs are present in the dog genome. But, interestingly, the dog also contains an additional 5 full-length genes; EMR2b, EMR2c, EMR2d, EMR4b and EMR4c; and 1 pseudogene GPR133b. These *Adhesions *GPCR genes seem to be specific for the dog lineage as they have not been found in other mammals studied [8,30,31]. We performed a phylogenetic analysis based on the 5 dog-specific EMR receptor sequences together with the dog, human, cow and opossum EMR1-EMR4 and CD97. The phylogenetic analysis was based on the transmembrane regions and the resulting consensus tree is presented in Figure 3. The dog and human one-to-one *Adhesion *receptor orthologs have an average protein sequence identity of 83% and this is higher than each of these species have to their mouse counterparts (Figure 2). GPR144, EMR2 and EMR3; which are full-length in human but pseudogenes in rodents; appear to be functional (are full-length) in dog. The gene sequences of BAI1, EMR2d, EMR4c, GPR123 and GPR124 are incomplete.

The *Frizzled *family is well conserved between dog, mouse and human having 11 orthologous receptor pairs and no species-specific genes in either species (Figure 2 and Additional file 1). A slight difference is observed for the rat *Frizzled *repertoire in which FZD10 appears as a pseudogene. The average amino acid identity is 96.9% between dog and human *Frizzled *orthologs. The gene sequence of dog FZD8 is incomplete.

The *Secretin *family has the same 15 members in human, dog, mouse and rat i.e. their repertoires are identical. The average protein sequence identity between dog and human *Secretin *family GPCR orthologs is 88.5% (Figure 2).

The group defined as *Other GPCRs *include 18 dog genes. One of these, GPR172A, which is present in human but missing in rodents, was found to be missing in the dog genome. Another gene, TMEM185B, which is a pseudogene in human but full-length in rodents, appears to be functional (is a full-length gene) in dog.

## Discussion

In this study we present the overall repertoire of non-olfactory GPCRs in dog and compared it with its counterparts in human, rat and mouse. Comparison of the dog and human GPCR repertoires, tabulated in Tables 2, 3, shows 12 GPCR genes that are only found in the dog genome. Moreover, 20 human GPCR genes were not found in dog while 4 human GPCR genes were found as pseudogenes in the dog genome. There are a variety of possible underlying reasons and consequences of why some receptors have been lost or duplicated in some species, but not affected in others. The general evolutionary explanation is that gene repertoires are altered in response to the adaptation of the animals to environmental factors such as the availability of food, disease, predators and appropriate habitat. Behavioural factors such as cooperativity, care of young, learning and social hierarchy also come into play. Receptors would be gained if gene duplication offers a functional advantage by allowing for an increased or altered expression (e.g. change in tissue distribution or expression level) of the protein or the gain of a new function (e.g. a different ligand). If the redundancy is physiologically insignificant gene duplicates are typically lost by pseudogenization.

The result of the dog genome provides interesting insight into the differential evolutionary pressures among the subgroups of GPCRs. The majority of the differences observed in this study (7 of 12 GPCRs present in dog but not in humans and 12 of 24 GPCRs present in humans but not found in dogs) are found in only four sub-groups; EGF-TM7 (epidermal growth factor GPCRs), MRGPRs (Mas-related GPCRs), TAAR (trace amine-associated receptors) and FPR (formyl peptide receptors). The TAAR family is known to be highly variable between the mammalian species. For example, the number of intact TAAR genes are 5, 15 and 22 in human, mouse and opossum, respectively [32]. In dogs, there are only 2 intact TAAR genes; TAAR4 and TAAR5. The olfactory system is also associated with high interspecies variation at the mammalian level [26]. Pseudogenes are common in the olfactory repertoire, a feature that may relate to its peculiar signalling system, based on an olfactory neuron that has to have a signalling neuron to allow its connection to the apparatus of perception. A relatively dysfunctional GPCR has relatively little consequence as a result. The strong variations in the repertoires of the TAAR family are consistent with a common engine of evolution- sensory perception. Another sensory system, that perceives sweet or umami tastes, is mediated by three receptors in human, TAS1R1-3 (members of the *Glutamate *family). In cats TAS1R2 is a pseudogene and is therefore not available to form a critical heterodimer with TAS1R3 and this has resulted in loss of the ability to sense sweet tastes [33]. Dogs, unlike cats, are known to have an appetite for natural sugars. This fact is supported by genetics as TAS1R1, TAS1R2 and TAS1R3 all have intact gene sequences in the dog genome and thus can encode functional proteins. In contrast another receptor family that senses bitter tastants, the Taste2 receptors, are fewer in dog than in many other mammals. The number of Taste2 receptors is 14 in dog, whereas the corresponding figures in human and mouse are 25 and 34, respectively [27]. The number or bitter taste receptors in a species is likely to correlate with exposure to environmental factors vital for survival as bitter taste is an indicator of poison. Looking at other sensory genes such as the cluster of opsin receptors, the dog, like rodents, is lacking the Red opsin (OPN1LW) gene, which is essential for normal color vision in human. This difference has however a more specific consequence as compared with changes in the other sensory gene repertoires.

There is a large interest in the *Adhesion *family receptors, many of which were recently discovered [34,35]. *Adhesion *receptors have unique configurations of functional domains within their N-termini and these are thought to play different physiological roles by mediating a variety of interactions with extracellular molecules. One group of *Adhesion *receptors, the EGF-TM7 GPCRs, are equipped with a variable number of epidermal growth factor (EGF) and calcium binding domains and are reported to be important components of the immune system [34]. In dog, two EMR2-like GPCRs have been reported previously [36] and here we present one additional EMR2 and two EMR4 gene duplicates. Moreover, we found additional EMR2 duplicates in cow (See Figure 3). The 5 dog-specific EMR receptors are here termed EMR2b, EMR2c, EMR2d, EMR4b and EMR4c. It has been suggested that EMR2 has a chimeric structure [36]. The seven transmembrane (7TM) segments of EMR2 are most similar to those in EMR3 while the EGF domains in EMR2 are almost identical to those in CD97 [36]. Interestingly, in our phylogenetic analysis based on the 7TM segments (Figure 3), EMR2 and EMR3 orthologs did not cluster together and instead receptor paralogs grouped together. This is in line with the previous hypothesis about chimeric gene structures in this group [36]. We find this pattern to be the same for the human, dog, cow and opossum receptors (Figure 3). The new genes that we found in dog provide additional evidence for the unique evolution of the EMR subfamily of *Adhesion *GPCRs that seem not only shuffle domains within the N-terminal region but also larger segments of the N-termini.

Dogs are commonly used as model organisms in toxicity and dose tests in drug development and it has been proposed that the immune system is more similar between dog and human, than between mouse and human [37]. The EGF-TM7 have a role in the immune system [20] and it is intriguing to speculate if the additional members in dog may give this species an immunologic advantage. Chondroitin sulphate is a native ligand for both EMR2 and CD97, which can also bind decay-accelerating factor (DAF/CD55). The EGF domains in the N-termini of CD97 have been suggested to be essential for DAF/CD55 binding [38,39] while several other *Adhesion *GPCRs also have N-terminal EGF domains that could compensate for the gene difference in the mammalian gene repertoire. Interestingly, and a bit surprisingly, the formyl peptide receptors, FPR1 and FPRL2, could not be found in the dog genome. These receptors are also believed to participate in immune responses and respond to a large number of various ligands [40]. FPRL1 however has an intact gene sequence and appears to encode a functional receptor. It is possible that FPRL1 could have taken over the functions of two missing receptors in dog, pending that the sequence homology corresponds to a shared function. The involvement of the FPR and EMR families in the immune system could also be indicative of an immunological selection pressure that affects diverse groups of receptors. In drug development it is crucial to select model organism with genetics closely reflecting that of human and a model organism might prove inadequate because of differences in the gene repertoire. A missing dog ortholog may cause difficulties in drug development because the preclinical studies always include at least one non-rodent species, usually dog. Dog is good for the assessment of toxicity and it is easier to spot the effect of a drug in dog and more analytical instruments can be used e.g. electroencephalography and impedance cardiography. Differences in the immune system could also be potentially important for toxicity testing of a candidate drug, when it is crucial to have complete and functional immune system.

There are several other differences between the dog and human genome that are mostly related to *Rhodopsin *GPCRs. The MAS-related GPCRs (MRGPRs) family (in the δ-cluster) shows large variation between humans and dog. Most of the MRGPRs are orphan receptors, but some of them have known native ligands, like β-alanin, BAM8-22, cortistatin and angiotensin 1–7 [41]. This family is also highly variable in rodents and it is reasonable to assume that each of these receptor families is under strong selection pressure that is very species dependent. Also the *Rhodopsin *family γ cluster has several differences between the compared species. The neuropeptide B/W receptor 2 (NPBWR2) was not found in dog and is absent also in rodents and chicken. The somatostatin receptor 4 (SSTR4) seems to be missing only in dog and all five somatostatin receptors (SSTR1-5) are present in both the human and rodent genomes. However, it needs to be noted that conclusions on the effect of genes not found in dog are somewhat preliminary as it is possible that genes could be missing due to incompleteness of the genome assembly.

Our extensive dog GPCR gene sequence searches and the manual curation of the coding domains have resulted in an improved dataset compared to what was previously available in the public domain. We compared our dog GPCR dataset to what is available in the NCBI non-redundant (nr) database and found that our dataset contains 28 exclusive and 43 modified/curated full-length gene sequences. 282 of our full-length dog GPCR gene sequence have identical entries in nr. The corresponding numbers for the 13 dog GPCR pseudogenes identified in this study are; 2 identical in nr, 8 not found in nr and 3 modified/curated compared to nr. The higher number and significantly improved quality of the dog GPCR repertoire presented here clearly illustrates the value of manual sequence curation and extensive sequence collation from the genomic sequence.

In summary, we have presented the overall non-olfactory GPCR repertoire in dog and analysed it in relation to the human, rat and mouse counterparts. We have identified new genes and established the relationships of orthologs and species-specific receptors. This study describes in detail gene losses or duplications of GPCRs in both dog and rodents and this information is useful for the selection of model organism as it affects how to interpret pharmacological results.

## Conclusion

We present the first overall analysis of the non-olfactory GPCR repertoire in dogs and compare this to the versions in mouse, rat and human. The receptor sequences have been manually curated to assure a higher level of completeness and quality. Our results show that the dog GPCR repertoire is more similar to that in human than rodents both with respect to the number of receptor family members and the sequence similarity of orthologs. The comparison of the GPCR repertoires revealed several examples of species-specific gene duplications and losses and these were described in detail both for dog and rodents. This information can be used to guide the selection of model organism as gene redundancies or absences can have crucial effect on the outcome of pharmacological experiments and how they should be interpreted.

## Methods

### Identification of dog Glutamate, Rhodopsin, Adhesion, Frizzled and Secretin GPCRs

#### BLASTN searches in the non-redundant database

The human *GRAFS *GPCR genes were used as queries in individual BLASTN searches [42] against the NCBI non-redundant database [43]. For each query, the accession numbers of the 10 first hits (representing orthologs, paralogs and homologs) were collected and a non-redundant list was obtained which was used to collect the sequences of the hits using fastacmd of the NCBI blast package.

#### Manual curation of predicted gene sequences

The dataset obtained from the above search contained many predicted genes and these were manually curated for incorrectly included or left out exons. Incorrect sequences were identified from pair-wise comparison with the human ortholog or most similar homolog. Missing exon sequences were obtained from BLAT searches in the dog genome assembly using the human protein sequence as a query. Because of the genomic complexity of the dog *Adhesion *GPCRs only their TM regions were checked and corrected. For other GPCR families the full-length sequences were curated.

#### TBLASTN searches in the dog genome assembly

Previously published datasets of the human, rat and mouse GPCR protein sequences were used as queries [30,44]. The query dataset was searched using TBLASTN against the May 2005 assembly of the dog genome with expectation cut-off value set to 1.0. The results were processed so that overlapping hits on the same strand were merged using a custom made Java program (available upon request). The chromosome coordinates of the merged hits were used to extract the corresponding sequences from the genome using fastacmd of the NCBI blast package. Each sequence was elongated upstream until the first start codon and downstream until the first stop codon using a custom made Java program (available upon request). The preliminary dataset was "cleaned" from non-GPCRs and GPCRs from other families by querying it against the Refseq database using BLASTN with the default settings. The criterion for including new GPCRs was that the first two hits belonged to the same GPCR family. Protein translations were obtained using transeq from the EMBOSS package [45] and from these the longest intact coding domain was extracted.

#### BLAT searches for missing dog orthologs

Missing dog orthologs were searched for using online BLAT [46]. Dog orthologs were defined as BLAT hits with higher sequence similarity to the human query than any other protein sequence in the Refseq database or in our dog GPCR repertoire dataset.

### Naming dog GPCR gene sequences

The dog sequences were named according to official Gene names of human, rat and mouse orthologs. Genes that are specific to dog, i.e. not found in other species, were assigned the name of the most similar paralog and appending a lower case single character suffix e.g. "EMR2b". Orthologous and paralogous gene relationships were initially assigned based on reciprocal BLAST searches of dog and human GPCRs and subsequently verified by phylogenetic analysis.

### Phylogenetic analysis

Human and dog GPCR sequences were divided into *Glutamate, Rhodopsin, Adhesion, Frizzled *and *Secretin *families. Phylogenetic analysis was performed for each of the groups. The 7TM helices (excluding loops and N-/C-termini) for amino acid sequences were determined from a multiple alignment with bovine rhodopsin. produced with ClustalW 1.81. The reason for using only the sequences of the helices is that these regions have family-wide similarity, whereas the similarity of other parts are typically subfamily-specific hindering broader comparison. The alignment was bootstrapped 100 times and 100 Maximum Parsimony trees were calculated with Phylip 3.67 [47]. The consensus tree of the 100 Maximum Parsimony phylogenetic trees was calculated with Phylip 3.67, plotted with Treeview and manually edited in CANVAS.

### GPCR family sequence similarity analysis

Human, mouse and dog GPCR protein sequences were divided into separate datasets for the *Glutamate, Rhodopsin, Adhesion, Frizzled *and *Secretin *families. Full-length amino acid sequences were aligned with ClustalW 1.81. The percentages of protein sequence identity were calculated individually for each orthologous pairs from human, mouse and dog and used to derive an overall average for each GPCR family.

## Abbreviations

**ADRA1B**, **ADRA1D**, **ADRA2A**: alpha adrenergic receptor; **AKC**: American Kennel Club; **BAI1**: brain-specific angiogenesis inhibitor 1; **BLAST**: Basic Local Alignment Search Tool; **BLAT**: BLAST-Like Alignment Tool; **DAF**: decay-accelerating factor; **DARC**: Duffy antigen receptor for chemokines; **DRD4**: dopamine receptor D4; **EGF**: epidermal growth factor; **EMR**: EGF-like module containing, mucin-like, hormone receptor-like; **FPR1**: formyl peptide receptor 1; **FPRL2**: formyl peptide receptor-like 2; **GABA**: gamma-aminobutyric acid; **GPCR**: G protein-coupled receptor; **KISS1R**: KiSS1-derived peptide receptor; **MAS1L**: MAS1 oncogene-like; **MRGPR**: MAS-related GPCR; **MTNR1B**: melatonin receptor 1B; **NPBWR2**: neuropeptides B/W receptor 2; **NPFFR1**, **NPFFR2**: neuropeptide FF receptor; **NPY2R**: neuropeptide Y receptor Y2; **OPN1LW**: opsin 1, long-wave-sensitive, Red opsin; **PTHR1**: parathyroid hormone receptor 1; **P2RY4**: pyrimidinergic receptor P2Y, G-protein coupled, 4; **RXFP4**: relaxin/insulin-like family peptide receptor 4; **SSTR4**: somatostatin receptor 4;**TAAR**: trace amine associated receptor; **TM**: transmembrane; **TMEM185B**: transmembrane protein 185B; **TRHR3**: thyrotropin-releasing hormone receptor 3; **V1R**: vomeronasal receptors type 1; **V2R**: vomeronasal receptors type 2.

## Authors' contributions

TH performed the phylogenetic analyses, identified some of the gene sequences and wrote most of the manuscript. SMF participated in the design of the study and wrote parts of the discussion. HBS and RF participated in the design of the study and contributed to the manuscript. DEG conceived the study, participated in its design, identified most of the gene sequences and contributed to the manuscript. All authors have read and approved the final manuscript.

## Supplementary Material

Additional file 1 **Comparative table of the *GRAFS *family receptors in dog, human, rat and mouse.** A table listing the dog (cf), human (hs), rat (rn) and mouse (mm) GPCRs of the *Glutamate*, *Rhodopsin*, *Adhesion*, *Frizzled *and *Secretin *families. Pseudogenes are marked with a "P" in the column to the right of the respective species. When several species- or lineage-specific duplicates (paralogs) exist the paralog with the highest sequence identity has been given as the primary ortholog whereas the other genes are present on separate rows in the table and without a counterpart in the other species. Click here for file

Additional file 2 **Dog GPCR amino acid sequences. Complete list of dog GPCR amino acid sequences and pseudogenes in FASTA format.** Click here for file
